# When iconicity stands in the way of abbreviation: No Zipfian effect for figurative signals

**DOI:** 10.1371/journal.pone.0220793

**Published:** 2019-08-07

**Authors:** Helena Miton, Olivier Morin

**Affiliations:** 1 Department of Cognitive Science, Central European University, Budapest, Hungary; 2 Max Planck for the Science of Human History, Minds and Traditions Research Group, Jena, Germany; City University London, UNITED KINGDOM

## Abstract

Zipf’s law of abbreviation, relating more frequent signals to shorter signal lengths, applies to sounds in a variety of communication systems, both human and non-human. It also applies to writing systems: more frequent words tend to be encoded by less complex graphemes, even when grapheme complexity is decoupled from word length. This study documents an exception to this law of abbreviation. Observing European heraldic motifs, whose frequency of use was documented for the whole continent and over two large corpora (total N = 25115), one medieval, one early modern, we found that they do not obey a robust law of abbreviation. In our early modern corpus, motif complexity and motif frequency are positively, not negatively, correlated, a result driven by iconic motifs. In both our corpora, iconic motifs tend to be more frequent when more complex. They grew in popularity after the invention of printing. Our results suggest that lacking iconicity may be a precondition for a graphic code to exhibit Zipf’s Law of Abbreviation.

## Introduction

### Zipf’s Law of Abbreviation

George K. Zipf's name is linked to two phenomena: the power law distribution of word frequencies, and the correlation that he observed between word lengths and word frequencies—often referred to as the « Law of Abbreviation », or « Brevity ». It states that shorter words tend to be more frequent than longer ones [1]. Zipf’s Law of Abbreviation (ZLA) has been documented in various communication systems, both human and non-human. In the animal kingdom, a negative relation between signal length and frequency of use has been found, for example, in dolphins [2], formosan macaques [3], bats [4] and, to some extent, common marmosets [5]. Among humans, several empirical studies have verified Zipf’s Law of Abbreviation with both spoken and written communication systems. A ZLA obtains for all the spoken human languages for which it has been tested. A ZLA for phonological word length obtains in American English, Croatian, Greek, Indonesian, Russian, Spanish and Swedish [5]. Other studies, using number of phonemes as a proxy for word length, also found a ZLA in Dutch, English, German and Swedish [6,7]. The same result holds when orthographic word length (for alphabetically written languages) is used as a proxy for word length, as evidenced by studies based on more than a dozen languages [6–8] and one based on 986 languages [9]. Although no conclusive argument has proven Zipf’s Law of Abbreviation to be universal, it is certainly rather ubiquitous.

Zipf’s original account suggests that this law of abbreviation results from a tradeoff between a pressure for efficiency (favoring shorter forms) and a pressure for communication accuracy (favoring redundancy and unique, longer, forms). In this account, an optimal solution is a form of variable-length coding (similar to Huffman coding [10]) which assigns shorter words to more frequent meanings, and longer words to less frequent meanings. This type of coding would thus optimize the production cost of communication. Since frequently employed words or vocalizations overwhelmingly tend to be less informative than more frequent ones [6], Zipf’s Law of Abbreviation makes communication more efficient, by calibrating the amount of signal information that a receiver needs to process (e.g., the length of a word), to the quantity of information contained in the signal (e.g., a word’s predictability). In this account, a « Principle of Least Effort » [1] is to be understood as the functional explanation underlying the negative relation between words’ lengths and their frequencies.

### Both processing and production costs may cause the Law of Abbreviation

The exact causes of Zipf’s Law of Abbreviation remain unclear, due to a persistent ambiguity in the notion of communication efficiency. On the emitter’s side, efficiency refers to the effort spent on producing a signal; on the receiver’s side, it relates to the costs of processing a signal. Production costs and processing costs are tightly correlated: long words tend to be effortful both to produce and to process. Yet, as sociolinguists have argued, processing effort is unlikely to be perfectly aligned with production effort, for two reasons at least [11–13]. First, emitter and receiver may not be motivated to communicate to the same degree. In some situations (compare, for instance, a mumbled confession to a security warning communicated loudly and clearly to distracted passengers), speakers do not care as much about being understood as listeners do: speakers have an incentive in reducing their production effort at the expense of the hearer’s processing effort. Second, there are situations where context provides information that does not need to be linguistically encoded with precision. Here again emitters may reduce their production effort, this time without a corresponding increase in processing cost on the receiver’s side, since missing information can be inferred from contextual cues.

This opens the way for at least two distinct interpretations of Zipf’s Law of Abbreviation, depending on what one considers to be driving it. In one version, frequent words are shortened to make them more efficient to process, in the other, shortening facilitates the processing of frequent words. Although both versions result in tightly overlapping predictions, they are not impossible to tease apart. Studies addressing this issue [6,14] show that a word’s information value (its likelihood of appearing given the verbal contexts where it occurs) is a better predictor of word length than is word frequency (which is strongly but not perfectly correlated with information value). These studies are consistent with an interpretation of ZLA where abbreviation is driven by processing costs, rather than production costs, since a word’s information value affects the hearer’s capacity to anticipate it, but not the costs of producing it. In most studies, however, the exact roles played by processing *versus* production costs in the Law of Abbreviation are not teased apart.

### The Law of Abbreviation in graphic codes

This uncertainty on the exact roles played by processing versus production costs makes graphic symbols particularly relevant to the study of Zipf’s Law of Abbreviation [15–19]. Graphic symbols like written letters or emblems consist of visual marks inscribed on an enduring support (unlike the gestures of sign languages) [20]. The balance of processing and production costs is arguably quite different for graphic symbols as distinct from spoken words or gestures. Graphic symbols can be produced once and be seen many times, in contrast with spoken words, which need to be produced every time they are heard (exception being made for recent recording technologies of no relevance to language evolution). Techniques of mechanical reproduction, from seal impressions to printing, bring down production costs even further. Additionally, visual processing is intrinsically more efficient than phonological processing [21]. Graphic symbols, contrary to auditory signals, do not require their recipients to process them on the fly and on line, which could limit the impact of an increase in processing costs.

Testing for ZLA in a corpus of graphic symbols requires finding some graphic equivalent for the length of vocal signals. Image complexity is similar to the length of vocalizations in one key respect: complex images are harder and more costly to produce and to process. Longer words (above 7 letters) require longer reaction times to be recognized out of context [22,23]. Similarly, more complex images take longer to be identified, and also occasion more mistakes [15,24–26]. This effect of complexity is robust to participants’ familiarity or experience with the images [24], and to levels of noise, overall contrast, or eccentricity in the visual field [16]. More complex shapes, like longer vocal signals, both require higher cognitive costs to be processed than their simpler or shorter analogues. Following a Zipfian logic, any communication system, vocal or graphic, should minimize its aggregate costs by reserving long or complex forms for infrequent symbols. This predicts that visual complexity would be lower for more frequent graphic symbols, in the very same way that more frequent signals tend to be shorter in other communication systems [18].

Case studies have documented such distributions in a particular type of graphic communication system: writing systems. Consider as an example the visual complexity of logographic Chinese characters for Mandarin. A proxy for complexity, in this case, is provided by the number of distinct strokes that a character contains: 一 (pinyin *yi*, one) has fewer strokes than 五 (pinyin *wu*, five), thus it is less complex. Frequently used Chinese characters tend to be simpler, consistent with Zipf's Law of Abbreviation [16]. Unlike alphabetically written words, the complexity of Chinese characters is uncorrelated with the length of the morpheme they represent (which is one syllable-long, with rare exceptions): the “law of abbreviation” observed for Chinese characters thus cannot be due to the length of the underlying vocalizations. The same argument can be made for Chinese characters as used within the Japanese writing system (*kanji*): here again a “law of abbreviation” is observed [17]. Finally, it is also observed for large-size syllabaries or alphabets [18,19]. These writing systems (at least Japanese, Chinese and Vai) have made extensive use of printing, showing that ZLA may obtain for signals with relatively weak production costs.

### European heraldry

An equivalent of Zipf’s Law of Abbreviation for graphic symbols thus looks plausible on theoretical and empirical grounds. We turned to European heraldry to test it. The coats of arms (hereafter simply “arms”) used by notable European families since the late Middle Ages provide us with a corpus of graphic symbols that is abundantly and accurately documented over several centuries. Arms were versatile symbols. They could come in all sorts of sizes and on any and all kinds of support, from painted banners to impressed seals, from hand-drawn armorials to wrought-iron door knockers. Their uses ranged from the ostentatious (e.g., in tournaments, on monuments) to the mundane (e.g. as marks of property) [27,28]. The most important sources are, for the medieval period, painted armorials and engraved seals, joined for later periods by printed armorials and ex-libris plates. Heraldic emblems were created by combining motifs from a standardized repertoire of motifs that shows great stability across time and space [27,28]. Heraldic arms, thus, are ideally suited to a computational treatment: the appearance of motifs on the coats of arms of individual families can be estimated with precision, as well as the occurrences of motif combinations [29]. In this respect heraldic motifs resemble the written words of a well-documented script.

In addition to the abundance of high-quality data, our decision to study heraldry was justified by several notable analogies and disanalogies between heraldic emblems and linguistic symbols (written or spoken), which would make the obtention of a ZLA anything but trivial— a strong confirmation of this phenomenon’s apparent universality.

### Why heraldry may be Zipfian

#### Ubiquity of Zipf’s Law of Abbreviation

Zipf’s Law of Abbreviation’s quasi-universality is the first reason we would expect it to apply to heraldic motifs: as developed above, Zipf’s law of abbreviation can be found in a large variety of communication systems, both for oral and written signals. The basic mechanisms that cause ZLA in spoken words and graphemes appear to be present in heraldry: symbols were produced to encode information—in this case, to identify a coat of arms as belonging to a given family—, at a non-trivial cost to the producers. The information conveyed by heraldic emblems could be more or less ambiguous, and makers of arms strove to maximize the distinctiveness of the emblems they designed [29]. Although spoken and written communication systems (including heraldry) differ on specific properties, pairing shorter or simpler signals with higher frequencies and longer or more complex signals with rarer frequencies is an optimal solution, both in terms of minimizing the production effort (Least Effort interpretation of ZLA), and in terms of maximizing the informativeness in relation to the processing cost for receivers. Visually complex emblems, like complex letters and longer spoken words, are more costly to produce and process than their simpler or shorter analogues.

#### The appeal of simple motifs

In addition to this crucial pressure for distinctiveness, heraldic emblems were also required to be æsthetically pleasing. The search for æsthetic appeal may push down the complexity of the most popular heraldic designs, due to the well-attested link between the ease of processing visual stimuli and their perceived beauty [30,31]. Shapes are seen as more appealing when they are easier to process in a variety of experiments that manipulate parameters with known links to visual complexity, such as asymmetry or noisiness. Visual complexity directly decreases the ease of processing a visual stimulus [26]. This possible link between a symbol’s success, its æsthetic appeal, and its visual simplicity, was a good reason to study heraldic emblems, since it is unlikely to obtain in other graphic codes, such as writing systems (where a letter’s frequency is chiefly driven by the frequency of the morphemes or phonemes that it stands for).

Heraldry also included, from its origins onwards, motifs varying in complexity from relatively simple forms to relatively complex ones. Although some very simple motifs (e.g., a pale) were deemed ‘honorable’ and reserved to the oldest noble families [28], such motifs were relatively freely adopted. Such an association of simple forms with prestige, if anything, should favor the success of simpler motifs and hinder the diffusion of more complex motifs, thus predicting to the emergence of a ZLA.

### Why heraldry may not be Zipfian

#### Production costs

Heraldic motifs also differ from linguistic symbols in ways that suggest ZLA may not obtain in their case. First, there are reasons to believe that production costs were particularly low for heraldic emblems as compared to writing. They were frequently used for public display, where a symbol is produced once to be seen many times. Written characters, as used in personal correspondence or regular account keeping, must be inscribed repeatedly and rapidly. Production costs were also dramatically reduced by techniques of mechanical reproduction. These include printing, but also (and arguably, more importantly) seal impressions, thanks to which one heraldic emblem could be engraved once and impressed hundreds of times. Both techniques were used for written letters as well, but at least as far as seals were concerned heraldry depended upon mechanical reproduction to a greater extent. As a consequence, one could afford to produce heraldic emblems slowly and painstakingly, while written symbols in most contexts had to be drawn in fast and effortless ways. Another consequence was an increased division of labor: relatively fewer people were involved in the production of heraldic arms, compared to written symbols.

#### Iconicity

The third major difference between heraldry and writing lies in the fact that heraldry makes a sharp distinction between iconic and non-iconic motifs, and includes both types of images. Iconicity is defined as a salient perceptual or structural resemblance between sign and object [32]. Iconic (or *concrete*) symbols are assumed to be *visually obvious*: they successfully figure real-world plants, animals, persons, or objects, in ways that are immediately transparent for an unacquainted viewer [33]. In contrast, abstract symbols represent information using graphical features that have no obvious relation to what they represent. Both types of motifs figured on arms that stood for lineages, the mapping between arms and lineages being arbitrary most of the time. In this sense, both could be called “iconic” in the technical, Peircean sense that there was no resemblance between a symbol (the arms) and its referent (the lineage) [34]. Yet the motifs that we call iconic differ from the non-iconic in that they directly depict a real-world object (a lion, a cup, a knight, etc.), independently of their heraldic meaning(s). A wealth of arguments supports the idea that abstract graphic symbols evolve from earlier (more) iconic depictions, including both semiotic experiments [35,36] and observations on writing systems [37]. For instance, although many of them begun as iconic signs, the figurative meaning of most Chinese character keys is either lost or beyond the uninitiated’s grasp [38]. By contrast, the rules of heraldic composition differentiate two categories of motifs. The “charges” (e.g. a lion, an eagle, a castle, etc.) can be placed anywhere on a coat of arms and they are overwhelmingly iconic; the “ordinaries” (e.g. a bend, a chevron, etc.) are not, and their location is constrained in various ways [39]. This distinction allows us to separate iconic from non-iconic motifs using the categories given by our sources. In the rest of the paper we simply refer to charges as “iconic motifs”, and to ordinaries as “non-iconic motifs” (see Methods and S1 File for more detail).

Iconicity may prevent the emergence of a ZLA. In order to successfully represent their real-world referent, concrete symbols include details enhancing the similarity between the symbols (or drawings) and the objects they are representing: for instance, symbols for bears would depict fur and other characteristics of actual bears. Concrete symbols have been found to be more complex than abstract symbols over a range of studies ([40] analyzing data from [41]; [42–44]). Concrete symbols also enjoy performance advantages over abstract symbols [44–46] in spite of their greater complexity: they are easier to recognize. For these two reasons, finding a graphic equivalent of Zipf’s Law of Abbreviation for heraldic motifs in addition to writing systems would be a strong indication of its universality.

### Cultural diffusion and the law of abbreviation

One last reason to study heraldry has to do with possible links between cultural diffusion and Zipf’s Law of Abbreviation. A written character’s frequency of use largely depends on the frequency of use of the underlying morpheme or phoneme. Heraldic motifs, in contrast, do not generally show such a dependency of their frequency on the frequency of what they represent. The frequency of the word “hedgehog” has much to do with the frequency of hedgehogs in the environment, but there is no equally direct link between the frequency of the hedgehog motif in heraldry and the frequency of hedgehogs, or that of families named “Hedgehog”. In other words, the frequency of heraldic motifs cannot generally be said to follow from semantic constraints, unlike the frequency of words. Rather, their frequency of occurrence reflects cultural diffusion, i.e., the selective borrowing of motifs resulting in their spread. The role cultural diffusion might play in ZLA is, at the moment, under-explored. Does frequency of use cause signals to become simpler, or on the contrary, do simple signals find more users? Both hypotheses are plausible. They are also not mutually exclusive. Use, especially in interactive contexts, tends to produce ZLA distributions of label lengths [47], while on the other hand, a signal’s brevity can lead such a signal’s frequency to increase, as the worldwide success of the word « OK » can attest [48].

Heraldry enables us to explore the impact of cultural diffusion in the long run, with two large corpora of European arms, one gathering arms dating from the late Middle Ages (c. 1200–1500) (“Clemmensen” [49]), the other gathering arms from the early modern period (c. 1600–1850) (“Renesse” [39,50]). This allows us to test how the cultural diffusion of motifs may impact a relation between motif complexity and frequency, but also how such a relation may evolve over time. In addition, the two corpus capture two different states of European heraldry, whose history can be seen as leading from relatively simple arms to more complex ones, partly (but not only, see below) because the simplest motifs were thought to be the preserve of the most ancient families, who chose their arms before others did [50].

### Goals of the study

The present study aims to (1) test for a ZLA in heraldic motifs over two corpora, one medieval, one early modern; (2) investigate whether—and to what extent—iconicity interacts with ZLA, and (3) explore the impact of cultural diffusion upon these dynamics.

**Fig 1:**
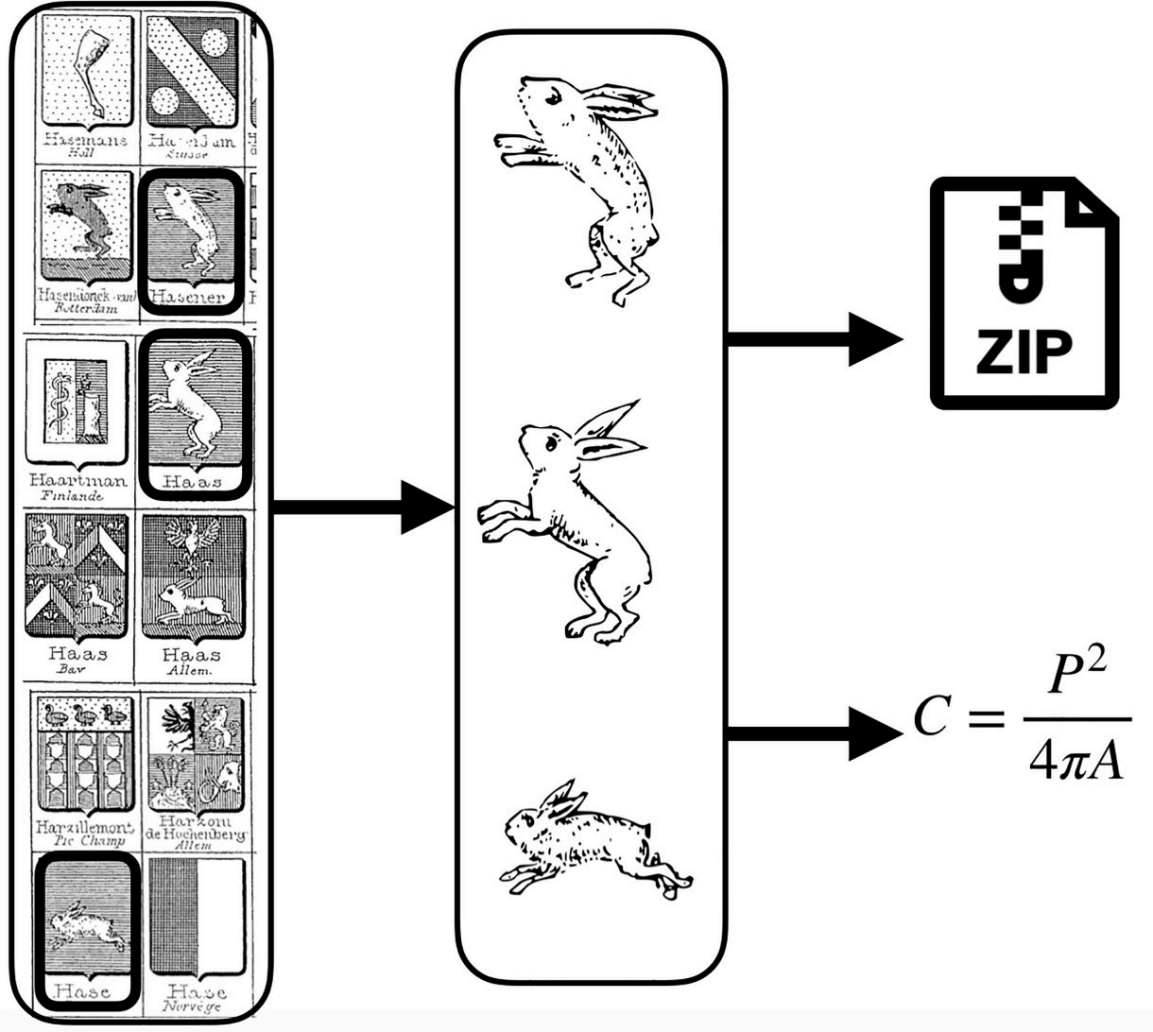
Visual complexity metrics derivation. Our complexity measures were obtained in the following way: (1) three arms were selected for each motif from our reference armorial [56], (2) pictures of shields were edited to obtain a picture of the motif on its own, (3) the edited pictures went through the Potrace algorithm to improve their quality (through vectorization), (4) they were zipped and the zip file’s size served as our measure of descriptive complexity, while their perimetric complexity was calculated using Mathematica. Finally, all the three measures from the three pictures obtained for each motif were averaged to get one reliable measure for each type of complexity for each motif in our inventory.

The complexity of graphic symbols can be measured in various ways. Of the multiple definitions that have been proposed for what makes a shape simple or complex (see [51] for a review), we focus on two measures from two distinct research traditions. The first one, Descriptive Complexity (DC), is based on Algorithmic Information Theory and uses the length of code required to store an image in optimally compressed form as a proxy for the image’s complexity. Here, it is obtained by compressing the picture files and using the size of the compressed file (in bytes). The second one, Perimetric Complexity (PC), starts instead from the image’s physical features. Some such measurements consider, for instance, the number of angles or edges in images, or their ratio [52,53]. Here we consider, instead, the image’s contour length compared to its inked surface [26,54] (see Materials and methods). Both these proxies for image complexity correlate negatively with ease of processing and performance for an array of tasks (see [26] for PC, [25] for DC). In order to have reliable estimates of heraldic motifs’ complexity, we used a compendium of more than 100 000 illustrated arms [55], from which we extracted three images for each of the motifs present in one or both of our corpora (Fig 1). All images used in the present study are available on the OSF depository associated to the project (https://osf.io/ykp37/).

## Materials and methods

In order to be able to test our predictions, we compiled (1) a list of motifs whose frequency and complexity we measure, (2) pictures to reliably measure the motifs’ complexity, and (3) frequencies (i.e., number of occurrences) for such motifs, on two corpora corresponding to two different time points: the Clemmensen (c. 1200–1500) and Renesse (c. 1600–1850) corpora—see Table 1 for more details on our sources and how they relate to each other.

**Table 1:** Our sources, how they relate to each other, their format, and which information was used from each one.

Source	Relation to other sources	Format	Information extracted
Rietstap	indexed by Renesse, illustrated by Rolland	Armorial (list of branch’s names and descriptions of their arms)	None directly
Renesse	indexes and classifies Rietstap’s armorial	Dictionary (list of branches organized by which motifs they bear)	Frequencies Motifs’ inventory (classification)
Rolland	illustrates Rietstap	Compendium (tables of illustrations)	Pictures of motifs
Clemmensen	None	Armorial (list of branch’s names and descriptions of their arms)	Frequencies

While Renesse provides frequency data for the early modern period, Clemmensen provides it for the late Middle Ages.

### Pre-registrations

We kept a complete research diary on the Open Science Framework ([removed for blind review]) where all analyses carried out were pre-registered and described. Pre-registration is an open research practice that consists in describing the research design and analysis plan as independently as possible from data collection [57]. The methods and analyses of this paper were pre-registered (recorded) in several waves.

### Sources

Our primary materials were Renesse's *Dictionnaire des figures héraldiques* [50], the *Armoiries des familles contenues dans l'Armorial Général de J*.*B*. *Rietstap*, by Victor and Henri Rolland, and Steen Clemmensen’s Armorial ([49], armorial.dk). Renesse [50] provides a motif-by-motif index of over 100 000 arms, indexing Rietstap’s *Armorial Général* [39], while the Rollands' compendium of arms [55] provides illustrations for over two thirds of those. Renesse provided a classification of motifs, which was used for both corpora, and frequency data for the Renesse corpus. Rolland provided the pictures of motifs we needed for our visual complexity measures (for both corpora). Finally, Clemmensen’s armorial provided us with frequency data for the Clemmensen corpus.

### Inventory constitution

A list of motifs corresponding to Renesse's classification was built taking Renesse's own subdivisions of his material as guide. Two aspects of motifs that were relevant for the author were not taken into account for classification: the orientation of a motif (i.e. whether the same motif is presented facing the left side or the right side of the arms), and the number of times that it is repeated. In other respects, we stuck as close as possible to Renesse's own descriptions. All further details and steps of sample constitution are reported in S1 File. Information on frequency and information on complexity were collected independently—i.e., the researcher and research assistants who collected the frequency data did not observe the visual complexity data, and vice versa.

Our classification of motifs between iconic and non-iconic motifs was directly built on Renesse’s inventory. Following a long-established taxonomy, his inventory makes a sharp distinction between certain categories: “charges”, which are any image that can be placed anywhere on the arms, and “ordinaries”, which includes both “pièces”, whose placement is constrained by rules, and “partitions”, which are divisions of the arms. Ordinaries are abstract, geometric shapes that do not represent a natural object in any detail (e.g., saltires, bends, lozenges). The subset of motifs they represent is referred to as non-iconic. By contrast, charges are essentially figurative motifs, representing mainly animals, plants and various artifacts, and the subset they represent are referred to as iconic.

### Visual complexity

All the complexity measures were taken as the average of three arms (i.e., three image files), selected among a standardized collection of thousands of drawings [55,56]—see S1 File for details on arms selection and image files preparation. Using three pictures for each motif allowed us to have robust estimates of the motifs’ complexity that would not depend on the specific picture chosen for each motif while still allowing to include a large number of motifs. We used two measures of complexity: perimetric complexity and descriptive (also known as algorithmic) complexity. Both complexity measures have previously been used in experimental investigations of cultural evolution [58].

Descriptive complexity measures are obtained using the potrace algorithm [59] on the .pnm files, and then compressing the obtained .eps file. The proxy for descriptive complexity is then the size in bytes of the compressed file: it offers an estimation of the length of the shortest computer program that (losslessly) produces the image. This measure of descriptive complexity is identical to the one used by Tamariz & Kirby [58] under the label *algorithmic complexity*. It is to be conceived of as an upper bound of a picture’s complexity, as (1) it adds header information—which was kept minimal using the same folder for all pictures, and standardized file names of the same length, (2) it only searches for a small set of simple patterns and patterns in particular block lengths, and (3) it is not a mode of compression optimized for images per se.

We measured perimetric complexity [26,60], defined as a ratio of inked surface to the perimeter of this inked surface. It is obtained, using Watson’s implementation [60], by taking the squared length of the inside and outside perimeters of a motif *P*, divided by the foreground area *A* and by 4π, i.e.: $PC=\frac{P^{2}}{4\pi A}.$ The measure was implemented in Wolfram (Mathematica), and applied after the pictures were processed using the potrace algorithm.

As stated in the pre-registration documents, and in order to avoid motifs whose complexity measures would be unreliable (because of excessive variation in their depiction), we set a threshold over which motifs’ occurrences were too variable to be comparable, such that motifs for which our set of three pictures had a standard deviations higher than this threshold were excluded from subsequent analyses. This threshold was pre-registered, and applied to both measures of complexity. It is defined as two standard deviations above the mean of standard deviations (calculated for each motif on the basis of three pictures, see Eq 1)

t=M(SD(d))+2*SD(SD(d)) Eq (1)

with *d* being the distribution of complexity scores in our dataset of motifs.

Applying this exclusion criterion did not change our results: the results obtained without applying the exclusion criteria are available in S1 File, and are very similar to the ones reported in the main text.

### Frequency measures

A motif’s frequency refers to the number of arms bearing the given motif, among all arms bearing only one motif in each of our corpora. Thus, we only consider motifs occurring alone, i.e., we counted the number of arms bearing the motifs of interest and nothing else. This allows us to have (1) exhaustive counts for both corpora, which (2) are associated with representative visual complexity measures: our visual complexity measures are taken on motifs occurring alone on arms, and so are our frequency measures. We thus avoid biasing our frequency or complexity measures by having either of them include arms in which motifs appear in combination with other motifs. We used two corpora, one made from medieval armorials and covering mostly the period 1200 to 1500, based on the work of Steen Clemmensen [61] (here called “Clemmensen”), and another constituted by us from J.B. Rietstap's armorial [39] as indexed by T. de Renesse's dictionary [50] (here called “Renesse”). That second corpus covers a longer period, until 1880, although most of the arms that are dated occur between 1600 and 1850. Both corpora concern themselves chiefly with the arms of families and individuals, with little to no coverage of civic heraldry, and cover a wide range of European territories. We do not know to what extent the two corpora overlap: some arms are likely to be present in both. In other respects the corpora differ widely, and do not provide the same metadata. Although they do not classify heraldic motifs in identical ways, and have different ways of counting arms and families, our inventory of motifs was applicable to both. Details on how frequency measures were obtained for each dataset are available in S1 File.

### Statistical analyses

None of the measures we analyzed (for both Clemmensen and Renesse datasets, and all variables, i.e., frequencies and both measures of complexity) were normally distributed (all ps p < .01 on Shapiro-Wilk tests). Hence, all statistical tests presented here are non-parametric (Kendall rank correlation tests, because of the presence of ties, which leads to inexact p-values in Spearman’s rank correlation test). All analyses were run in R [62].

## Results

### Correlation between measures of complexity

**Fig 2:**
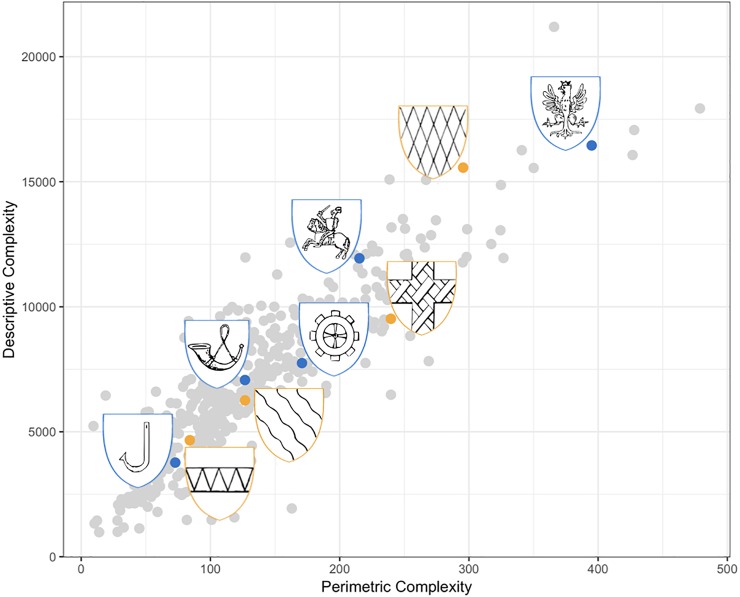
**Relation between perimetric (x-axis) and descriptive (y-axis) complexity**, with examples of iconic (blue frame) and non-iconic (yellow frame) motifs (total n = 447 motifs).

Based on 447 motifs, our two measures of visual complexity, descriptive and perimetric, were highly correlated, r_τ_ = .69, p < .001, 95% CI [0.657, 0.725]. See Fig 2 for illustrations of heraldic motifs of different visual complexity.

### Iconic motifs are more complex than non-iconic motifs

**Fig 3:**
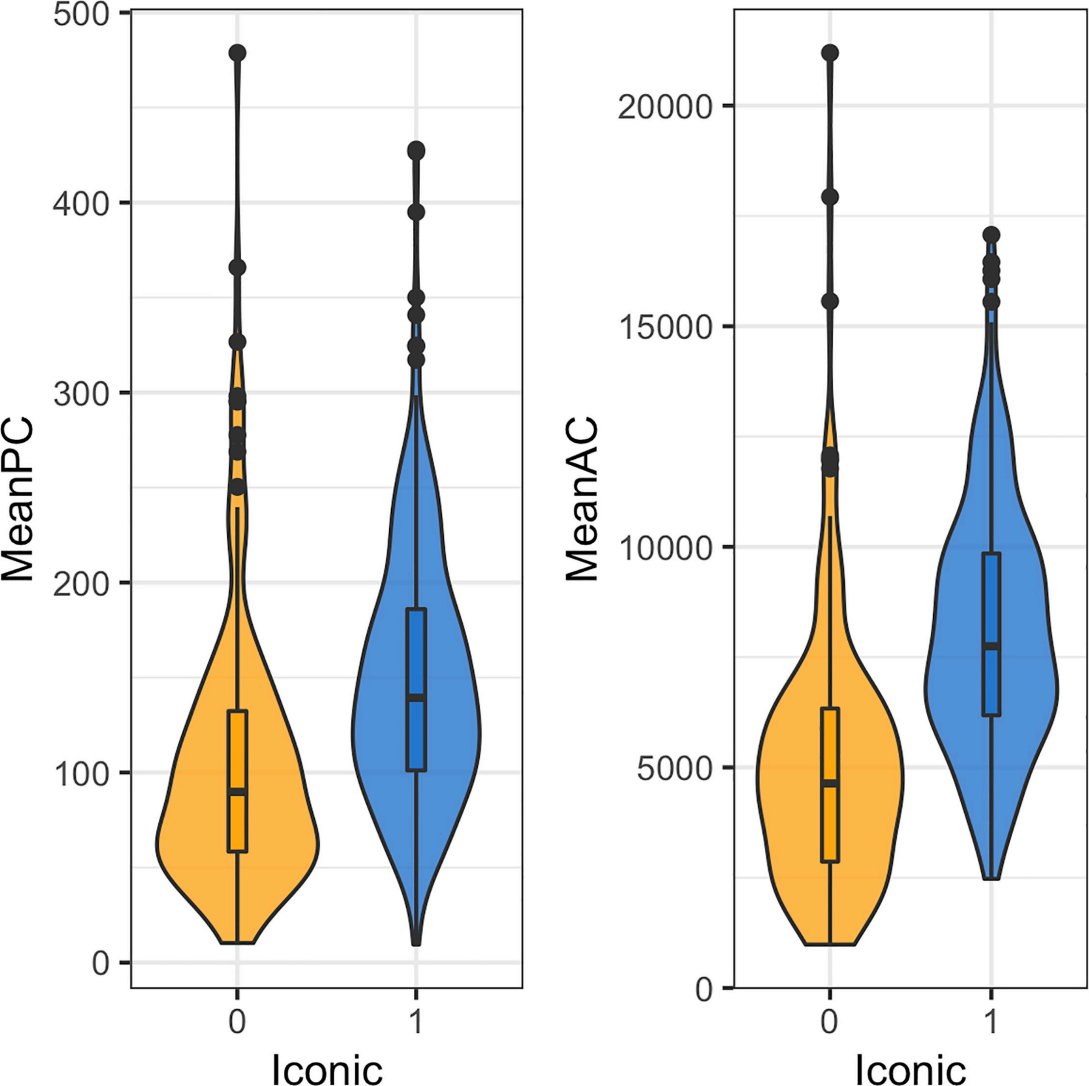
Distribution of iconic (blue) and non-iconic (yellow) motifs’ complexity scores, perimetric (left) and descriptive (right).

Previous experimental studies have interpreted decreases in complexity as indicative of a loss of iconicity [35]. Our study confirms that iconicity indeed tended to be associated with higher complexity: in our sample, iconic motifs (*N* = 295) had higher complexity than non-iconic motifs (*N* = 152), both perimetric (U = 32160, p < .01) and descriptive (U = 36110, p < .01), see Fig 3.

### No overall Zipf’s law of abbreviation

**Fig 4:**
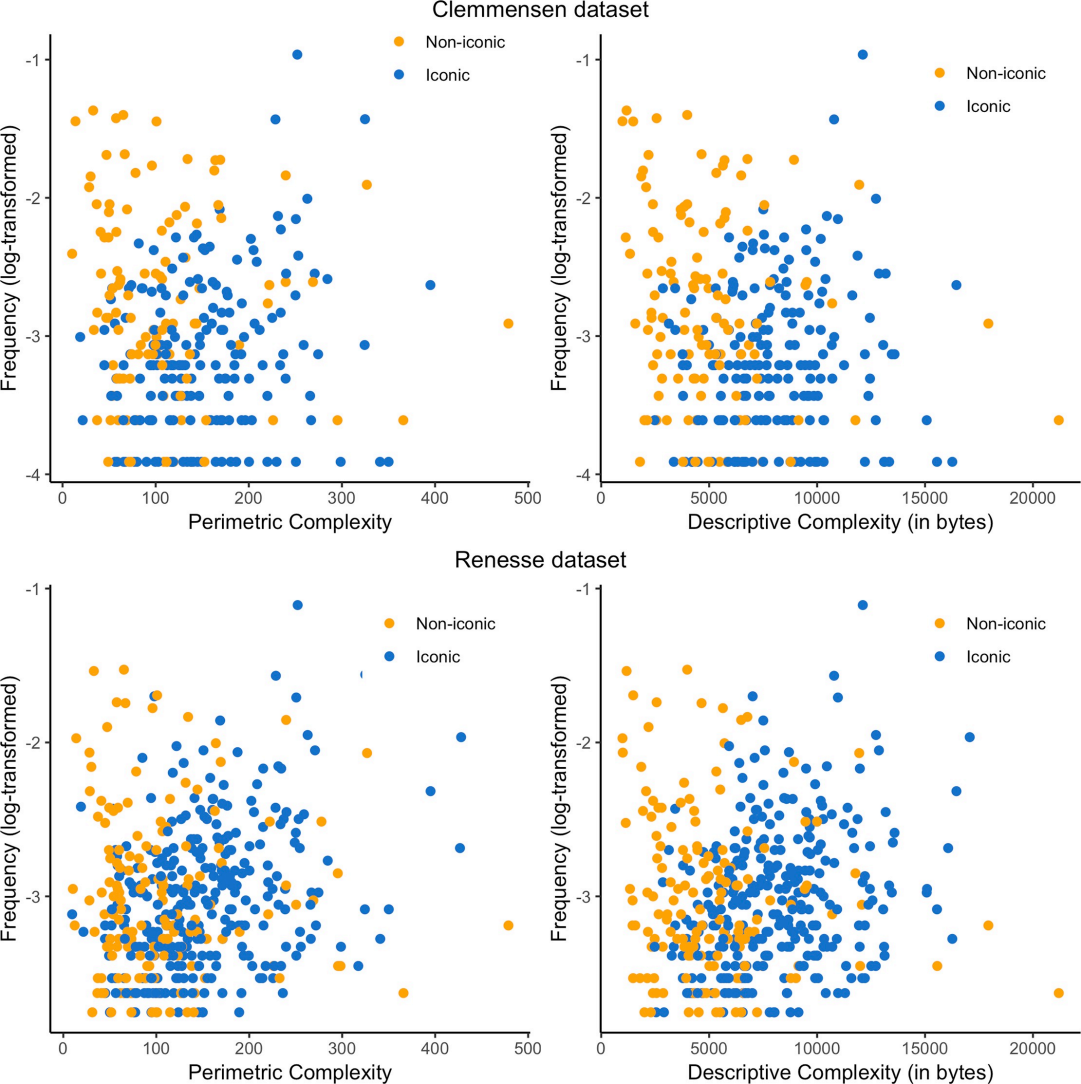
**Frequency (log-transformed) as a function of perimetric complexity (left) and descriptive complexity (right), for both corpora (Clemmensen above, Renesse below).** Color indicates iconicity—i.e., iconic motifs are in blue and non-iconic motifs are in orange.

For the Clemmensen corpus (296 motifs, total *N* = 8124 arms), we failed to conclusively observe a Zipfian correlation (see Fig 4). On the one hand, more frequent motifs also tended to be less complex when measured by descriptive complexity (r_τ_ = -.09, p = .018, 95% CI [-0.177, -0.011]). On the other hand, there was no significant correlation between perimetric complexity and frequency (r_τ_ = -.02, p = .633, 95% CI [-0.103, 0.065]).

Over the Renesse corpus (447 motifs, total *N* = 16991 arms), contrary to what ZLA predicts, more frequent motifs were also visually more complex. More precisely, there was a weak correlation between frequency and descriptive complexity (as evidenced by a Kendall rank correlation between frequency and descriptive complexity r_τ_ = .08, p = .008, 95% CI [0.02, 0.149]), and a stronger relationship between frequency and perimetric complexity r_τ_ = .12, p < .001, 95% CI [0.058, 0.186].

### No relation between complexity and frequency for non-iconic motifs

On the Clemmensen corpus, non-iconic motifs (114 motifs, *N =* 4795 arms) tended to be more frequent when they were less complex, similarly to ZLA’s predictions, but only when measured by descriptive complexity (r_τ_ = -.14, p = .035, 95% CI [-0.27, -0.001]). This correlation was not observed when measured with perimetric complexity (r_τ_ = -.08, p = .233, 95% CI [-0.211, 0.058]). For the Renesse corpus (152 motifs, *N =* 6529 arms), there was no correlation between frequency and perimetric complexity (r_τ_ = -.03, p = .534, 95% CI [-0.147, 0.078]), and only a trend for simpler motif to be more frequent, when using descriptive complexity (r_τ_ = -.10, p = .07, 95% CI [-0.211, 0.011]).

### Emergence of a ‘reverse’ ZLA for iconic motifs

Within the Clemmensen corpus, iconic motifs (182 motifs, *N =* 3329 arms) tended to be more frequent when they were more complex when measured by perimetric complexity (r_τ_ = .12, p < .05, 95% CI [0.021, 0.226]), but not when measured by descriptive complexity (r_τ_ = .08, p = .14, 95% CI [-0.028, 0.18]). By contrast, the Renesse corpus showed clearer results: iconic motifs (295 motifs, *N =* 10462 arms) showed a positive correlation between complexity and frequency (for both perimetric, r_τ_ = .22, p < .001, 95% CI [0.143, 0.291], and descriptive complexity, r_τ_ = .18, p < .001, 95% CI [0.108, 0.253]).

### Iconicity and complexity both predict increases in frequency

The proportion of iconic to non-iconic motifs significantly increased between our medieval corpus (Clemmensen) and our early modern corpus (Renesse), from 0.41 to 0.62 (binomial test: p < .001). This change in favor of iconic motifs was driven by iconic motifs’ frequencies increasing more than that of non-iconic motifs: when comparing the change in frequency for iconic and non-iconic motifs, iconic motifs (Mdn = 0.00035) increased significantly more than non-iconic motifs (Mdn = -0.00057, U = 16004, p < .001).

The more visually complex a motif was, the more likely its frequency was to increase between the two corpora, the medieval and the early modern, both when using perimetric complexity (r_τ_ = .16, p < .001, 95% CI [0.076, 0.242]), and descriptive complexity (r_τ_ = .22, p < .001, 95% CI [0.144, 0.297]) based on the 296 motifs present in both corpora.

**Fig 5:**
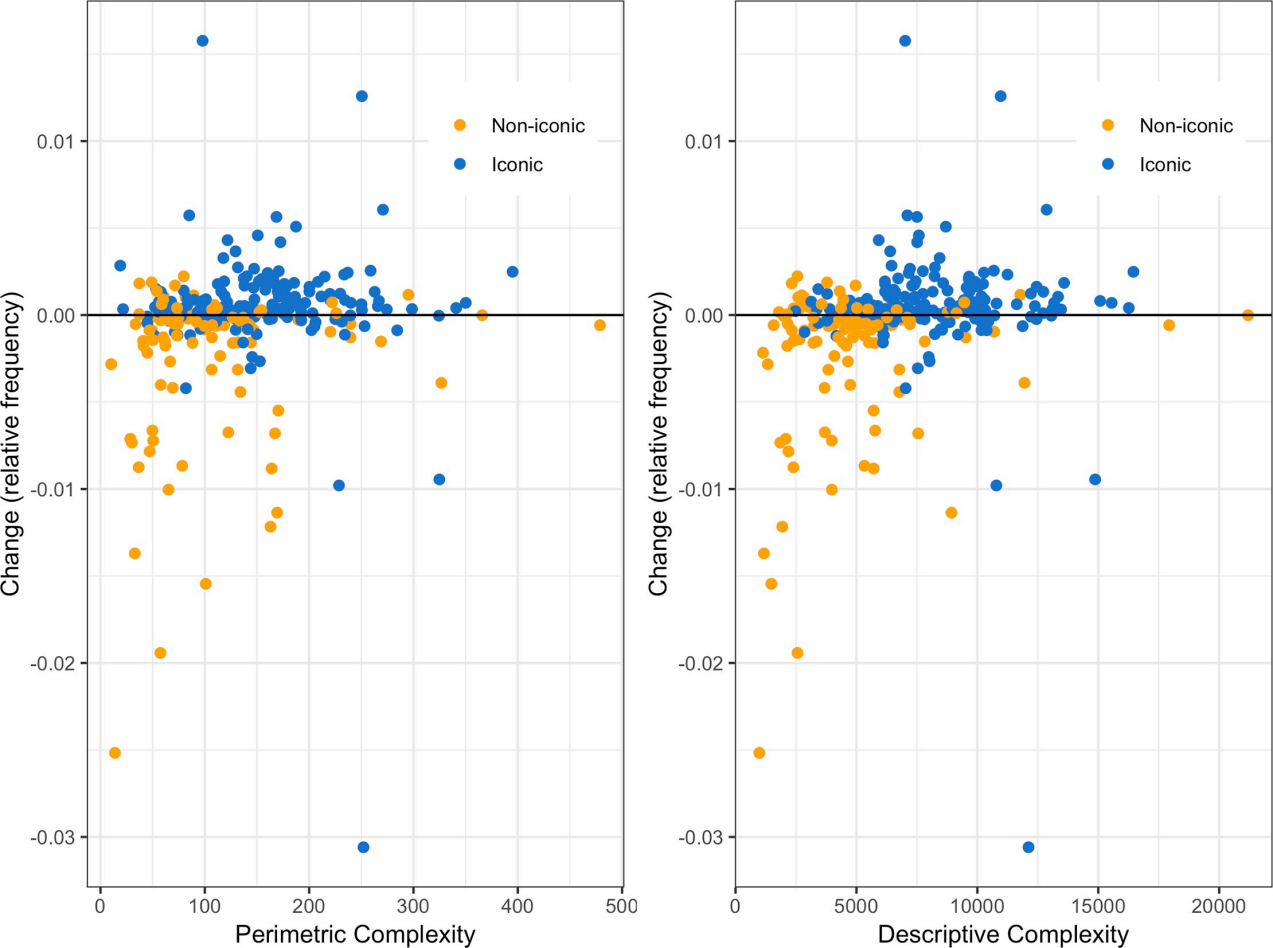
**Changes in frequency between our medieval corpus (Clemmensen) and our early modern corpus (Renesse) as a function of perimetric (left) or descriptive complexity (right).** Iconic motifs are represented in blue, and non-iconic motifs in orange. The horizontal line (y = 0) indicates no change in relative frequency between Clemmensen and Renesse. Points above the line represent motifs that increased in frequency in our early modern corpus compared to our medieval corpus.

The effect of visual complexity on changes in frequency differed for iconic as compared to non-iconic motifs (see Fig 5). On the subset including only iconic motifs (n = 182 motifs), there was a correlation between changes in frequency and perimetric complexity (r_τ_ = .11, p = .034, 95% CI [0.005, 0.206]), but not between changes in frequency and descriptive complexity (r_τ_ = .08, p = .108, 95% CI [-0.011, 0.172]). On the other hand, there was no effect of visual complexity on changes in frequency for non-iconic motifs (n = 114 motifs): there was no correlation between changes in frequency and complexity, neither for perimetric complexity (r_τ_ = .03, p = .590, 95% CI [-0.105, 0.173]), nor for descriptive complexity (r_τ_ = .09, p = .161, 95% CI [-0.049, 0.227]) on this subset.

## Discussion

Our study has three main results. First, we failed to observe a consistent Zipfian Law of Abbreviation in our two corpora. Frequent motifs were simpler in our late medieval corpus, but only weakly so. Motif complexity and frequency showed a robust correlation in our early modern corpus, but it went in the direction opposite to our prediction: frequent motifs were more complex, not less. Second, iconic and non-iconic motifs did not exhibit the same relationship between motif complexity and frequency. More complex iconic motifs were more frequent than less complex ones in both corpora, showing a reverse Zipfian effect, especially for our early modern corpus. By contrast, non-iconic complex motifs did not show this positive effect of complexity on frequency. Third, the present study documented the successful cultural diffusion of iconic motifs over abstract ones: the frequency of complex iconic motifs increased more than the frequency of simpler or non-iconic motifs between our late Middle Ages and early modern corpora. Zipf’s Law of Abbreviation, although frequently described as powerful and ubiquitous, fails to obtain for heraldic motifs.

Some previous empirical studies also failed to find Zipfian correlations. In animal behavior, there have been at least two cases in which communicative behavior failed to fulfill a ZLA: common ravens ([5], analyzing data from [63]) and golden-backed uakari vocalizations [64]. These remain rare examples, confined to non-human animals. Our results differ from these in that they do not merely suggest adding heraldic motifs to the list of cases in which we fail to observe a ZLA: they also show that the law can be reversed, at least for graphic symbols.

We now discuss these results through two major questions. Why does heraldry differ from other systems of symbols? Why do our two corpora differ to such an extent that complexity and iconicity are linked to frequency in the later one, but not the earlier? We consider and discuss four mechanisms that could be relevant to both questions. The first two, processing costs and production costs, plausibly influence ZLA for all types of signals, not just heraldry. The second concerns two factors peculiar to emblems, as distinct from spoken or written language: diffusion dynamics and iconicity.

### Processing and production costs

*Processing costs* might differ between written symbols, that exhibit ZLA, and heraldic emblems, that do not, since most written messages must be read at a faster pace than is required for emblems. This might weaken the pressure to simplify frequent symbols and explain why ZLA does not consistently obtain with heraldic symbols. However, this cannot explain the differences we observe between the Clemmensen and Renesse corpus.

*Production costs*. As argued in the introduction, the costs of producing heraldic symbols arguably differ for heraldic emblems compared to spoken language, and (to a lesser extent) to written symbols. Being destined for public display in durable formats, unlike private letters or spoken words, coats of arms were a type of symbol one could afford spending much effort on, in contrast with spoken, or even written, words, that have to be frequently produced anew. This decrease in production costs was compounded by the importance of heraldic seals, and, for the Renesse but not the Clemmensen corpus, by the advent of printing. Printing in itself is not sufficient to cancel ZLA, as ZLA is found in Chinese or Japanese written characters, two systems that have used printing for centuries [15,17]. However, it could be a facilitating factor. Processing costs thus provide a plausible, if incomplete explanation for the absence of a consistent ZLA in our two heraldic corpora, and for its reversal in the Renesse corpus. If right, this interpretation would imply that the principle of least effort at work in ZLA is (in this case at least) chiefly driven by production costs, not processing costs, in contrast with popular accounts of ZLA [6,14]. Note that production costs do not, by themselves, explain why the reversal of ZLA should be specific to iconic motifs.

### Diffusion dynamics and iconicity

This leads us to two distinct differences between heraldry and other graphic communication systems: the inclusion of iconic and non-iconic motifs, and the fact that the frequency of heraldic motifs depends mainly on their cultural diffusion (whereas the frequency of words is linked to how frequent their referents get mentioned in speech, whilst the frequency of letters depends on that of the phonemes or morphemes that they encode). Iconic motifs and their cultural diffusion were shown to play an important role in reversing ZLA for both our corpora. Iconic motifs tend to be of higher complexity than non-iconic motifs, both in this study and in others (e.g. [40]) as they are depicted with more detail, enhancing their resemblance with the element they are depicting. This, in turn, suggests that what causes our reverse ZLA may have to do with pressures favoring the diffusion of iconic motifs.

Why do our two corpora differ to such an extent that complexity and iconicity are positively correlated to frequency in the later one, but not in the earlier? Asking this is tantamount to asking what caused iconic motifs to spread to a much larger extent than abstract ones. One reason could be a quirk of early heraldic history: the simplest abstract motifs (e.g. one bend, a pale) were reputed to be “honorable” and reserved for the oldest nobility [28,39]: later-comers would need to make do with more variegated designs. True as this may be, this does not explain our results. The range of simple abstract shapes allowed by the rules of heraldry was in fact much broader than the set of “honorable” motifs, yet many possible designs did not find any adopter; and even “honorable” motifs were copied dozens of times [29]. We cannot assume either that complex, iconic motifs were chosen because simpler, non-iconic motifs were no longer available: some of them, like the lion, were popular from the very start. The variety and evocative strength of iconic motifs made them supplant abstract patterns in other graphic codes, for instance the seal marks of ancient Mesopotamia [65]. A more likely cause for the spread of iconic designs is thus, in our view, the printing press and the family of mechanical reproduction techniques that revolved around it, including etchings and lithography. Printing made it much easier to reproduce complex pictures reliably on multiple supports, and to diffuse complex, standardized motifs across distances. It marked a decisive break between the visual culture of the late Middle Ages and early modern periods (as witnessed, for instance, by the massive popularity of engravings). The introduction of mechanical reproduction techniques, by decreasing the production costs of complex iconic motifs would have driven the ‘reverse’ Zipfian effect we observed in our results, with more frequent motifs also being more complex.

Our results on heraldic motifs can be reconciled with the ubiquity of Zipf’s law of abbreviation, not only in vocal communication, but also in writing systems. The law obtains, not only in the overwhelming majority of vocal communication systems, but also in those writing systems where a character’s frequency is entirely decoupled from the length of the morpheme that it stands for. Yet these writing systems are devoid of iconicity. We argued that iconicity, aided by cultural diffusion and a change in production costs, stands in the way of abbreviation in the case of heraldic motifs. Lacking (or, at any rate, losing) iconicity may be a precondition for Zipf’s Law of Abbreviation to emerge in a graphic tradition.

## Supporting information

S1 File Supporting information. This document contains a guide to the materials available on Open Science Framework, basic heraldry vocabulary, additional methodological details, and results of the analyses without applying the exclusion criterion.
(DOCX)
