# Supplementary material for: When iconicity stands in the way of abbreviation: No Zipfian effect for figurative signals
Source: PLoS One. 2019 Aug 7;14(8):e0220793. doi: 10.1371/journal.pone.0220793 (PMC6685622; doi:10.1371/journal.pone.0220793)
Supplement: S1 File — This document contains a guide to the materials available on Open Science Framework, basic heraldry vocabulary, additional methodological details, and results of the analyses without applying the exclusion criterion. (DOCX) [file pone.0220793.s001.docx]

Supporting Information for

*When iconicity stands in the way of abbreviation*

**Contents**

[1. Guide to the OSF materials 1](#_Toc529300081)

[2. Basic heraldry vocabulary 2](#_Toc529300082)

[2. Additional methodological details 2](#_Toc529300083)

[2.1. Inventory constitution 2](#_Toc529300084)

[2.2. The iconic / non-iconic distinction in heraldry and in our data 3](#_Toc529300085)

[2.3. Shields selection 4](#_Toc529300086)

[2.4. Pictures’ preparation 4](#_Toc529300087)

[2.5. Frequency measures in the Clemmensen corpus 4](#_Toc529300088)

[2.6. Frequency measures in the Renesse corpus 6](#_Toc529300089)

[4. Results of analyses on the datasets without applying the exclusion criterion 6](#_Toc529300090)

(All bibliographic references refer to the main paper’s References list.)

# **1. Guide to the OSF materials**

The data files (dataClem.csv, and dataRenesse.csv), metadata (metadata_Zipfpaper.rtf**)** and R script (ZipfPaperScript.R) necessary to replicate all the findings presented in the manuscript are available here: [https://osf.io/ykp37/](https://osf.io/ykp37/?view_only=574c0052d159473d94485d2777ed5e9b)

We also pre-registered all the work that went into this project on the Open Science Framework’s website, where a complete, public record of the research process can be accessed. We release all the registrations and reports relevant to this study**.** The successive reports allow a complete picture of the research process involved in producing the study and results reported in the manuscript. All documents can be freely consulted on the Open Science Framework at the given URLs. They include:

- **zipf1-2016-08-03 (**<https://mfr.osf.io/render?url=https://osf.io/2fbns/?action=download%26mode=render>**)**: First pre-registration of this study^[[1]](#footnote-1)^. It presents the rationale of the study and the study plan, including possible follow-ups.

- **zipf2-2016-10-06 (**<https://mfr.osf.io/render?url=https://osf.io/tq97x/?action=download%26mode=render>): This document explains how we built our two corpus and measured the motifs’ visual complexity,with a description of our exclusion criteria (both pre- and post-measurements) and additional methodological details on the measurement of complexity, including the editing of motif pictures.^[[2]](#footnote-2)^

- **zipf3-2017-10-23 (**<https://mfr.osf.io/render?url=https://osf.io/42ser/?action=download%26mode=render>**):** This file reports a few departures from the initial registered study plan (due to characteristics of the materials used), additional exclusions, a short erratum correcting a paragraph from a previous registration, and the correlation between the two visual complexity measures that were used. It also registers the next version of the study^[[3]](#footnote-3)^, which is the one we report in this manuscript.

- **zipf4-2018-04 (**<https://mfr.osf.io/render?url=https://osf.io/jms8z/?action=download%26mode=render>**)**: This report includes results for the following: (1) whether frequency distributions (in both the Renesse and the Clemmensen datasets) followed scale-free distributions, (2), whether the motifs’ frequency was negatively correlated to visual complexity (i.e., what would have been predicted by a law of abbreviation), and (3) whether this correlation applied equally to both iconic and non-iconic motifs. It also states which files those results are based on. Finally, this report also stands as the registration for additional exploratory analyses that the results called for (mainly pertaining to the differences between the Renesse and Clemmensen datasets). Be aware that some of the results reported there were actually incorrect due to a small mistake in the R code (use of the wrong ‘Frequency’ variable). The correct results are the ones included in the manuscript.

**- zipf5-2018-05-22(**<https://mfr.osf.io/render?url=https://osf.io/672qz/?action=download%26mode=render>**):** Our latest report includes results for the following additional exploratory analyses: (1) whether there is a significant change in the proportion of iconic to non-iconic motifs between the two datasets, (2) whether complex motifs became more frequent and simpler motifs less frequent, independently of their iconicity, and finally (3) whether differences between Renesse & Clemmensen depend on the new motifs appearing in Renesse but not in Clemmensen.

# **2. Basic heraldry vocabulary**

| **Term** | **Definition** |
| --- | --- |
| Arms | Equivalent of coat of arms or shield. |
| Armorial | A collection of branches (part of families) names, with descriptions of their arms (“blasons”). |
| Motif | Images that can appear on arms. |
| Charge | Iconic motif, can be placed anywhere on the shield, roughly corresponds to “meuble” in heraldic French. |
| Ordinary | Abstract motif. Includes “pièces”, whose placement is constrained by rules, “partitions”, which are divisions of the shield, and “rebattements”, geometric patterns covering all or part of the arms. |
| Tincture | Ways in which any part of the arms can be colored, divided between colors, metals and furs. Furs (Hermine, Vair) are patterns, whereas colors and metals are plain colors. |

# **3. Additional methodological details**

We here present additional methodological details about data collection. They list all methodological decisions that were made related to processing our original (historical) materials.

## **3.1. Inventory constitution**

A first list of motifs was constituted from the general index of Renesse’s seven-volumes compilation. The index was text-captured and rearranged to yield a list of motifs. From this list of motifs, we made a series of deletions, for the following reasons:

- Removing duplicates (i.e., motifs that Renesse seems to have mentioned twice by mistake);
- All “extraordinaires” and “divers/diverses” items were removed, as they represent miscellaneous, quaint, or heterogeneous motifs not clearly standing for a well-formed category;
- The tinctures (red, blue, yellow, etc. — corresponding to gules, azur, or, etc. in heraldic parlance) were removed;
- Categories which were referred to interchangeably were merged into one;
- Chapter headings, which referred to the general theme of the chapter’s motifs, rather than to motifs themselves, were removed;
- Motifs that were not present alone on at least one otherwise empty shield (i.e., never present “in isolation”), were removed;
- Motifs that occurred in isolation, but only in groups of two or more repetitions, were removed;
- Geometric motifs occurring in groups of varying size were removed;
- Motifs that only occurred accompanied by other, different motifs, were removed.

Additionally, a few items were present inside Renesse’s volumes but not in the index were added to the list of motifs. Particular additions or deletions were reported in the Open Science Framework reports at each stage.

A second step consisted in cross-checking the list obtained with Renesse’s sections entitled “armes complètes”. For each motif listed in his index, Renesse, when possible, devotes a section to an exhaustive list of all the arms bearing the motif in question and nothing else. At this stage, items were classified into two groups: the charges (“meubles”, referred to as “iconic” motifs in our paper) on one side, the “pièces, partitions, rebattements, émaux” on the other (referred to as “non-iconic” motifs in our paper).

A third and last step required us to discard all the motifs that were represented by two arms or less, as the complexity measures were taken as the average of three shields. This happened either when the motif was featured on less than thee shields in total, or when the motif didn’t occur on its own on three shields (i.e., occurred only in combinations).

## **3.2. The iconic / non-iconic distinction in heraldry and in our data**

Renesse’s inventory, following a long-established taxonomy, makes a sharp distinction between certain categories: “charges”, which are any image that can be placed anywhere on the arms, on one hand, and “pièces”, whose placement is constrained by rules, or “partitions”, which are divisions of the arms. The English term “ordinaries” covers both “pièces” and “partitions” (Fox-Davies 1900). Ordinaries are abstract, geometric shapes that do not represent a natural object in any detail: saltires, bends, lozenges, etc. The subset of motifs they represent is referred to as non-iconic. By contrast, charges are essentially figurative motifs, representing mainly animals, plants and various artifacts and the subset they represent are referred to as iconic.

## **3.3. Shields selection**

Three shields were collected among the Rolland’s compendium of illustrations, for each motif. They were selected in the following way: (1) the shield bore the motif of interest and nothing else, and (2) shields where the motif is tinctured with white were given priority, unless the motif was tinctured with black 90% of the time or more. When there weren’t enough white motifs, the next preferred tincture was yellow. This constraint made it easy for us to process the pictures: motifs were edited to remove the tincture markings added by the illustrators to their designs. These markings were added by the Rollands to stand for tinctures, i.e., colors. White was coded with no marking, while yellow had the simplest of markings (dots). Finally, due to the structure of the source, choosing based the alphabetical order of the owners' name would have introduced a confound. Hence, the shields, as long as they satisfied the two previous criteria, were picked randomly.

## **3.4. Pictures’ preparation**

All pictures underwent a very light resizing to match a fixed 309 x 400 pixels template, which was able to accommodate all the shields that we selected. This resizing also made the various shields more easily comparable and compensated for irregularities in the original material. All shields also got their border erased, so that complexity measures could bear on the motifs themselves, and would not bear any additional noise due to irregularities in the borders’ printing. After editing, pictures were saved as .pnm files, and then had the potrace algorithm applied to them (Selinger, 2003).

## **3.5. Frequency measures in the Clemmensen corpus**

Our measures of frequency were based on Steen Clemmensen’s extensive database “armorial.dk” (version 12). We counted only the arms where the focal motif occurs in isolation: in one exemplar, and not accompanied by anything else.

Within this database, our measures were based on the table of branches, i.e., versions of a coat of arms possessed by a family, and presents a list of arms characterized by their design and the family or sub-lineage that carried them. This table includes 31 691 branches over 20 606 distinct families. Branches differ from families because a given family often came to possess various arms in the course of its history, either because the family branched out into distinct lineages, or because it decided to change the design of its arms.

Starting from the 31 691 branches listed by Clemmensen, were removed (1) all entries referring to mythical or heroic characters (corresponding to “MarchedArmes = _HERO”), and (2) all entries with no available arms (30 928 entries left).

Manual searches were then carried on both the English and the French versions of the list of arms, following criteria mimicking Renesse’s classification as closely as possible. To ensure that our counts included only arms featuring the motif in isolation, the following were discarded:

- Arms mentioning two motifs (or more), or repetitions of one motif;

- Arms mentioning any kind of accessory, prop or support not part of the original motif.

This includes (1) a mount, a hill or a terrace when a motif is poised on it, (2) any non-standard decoration accompanying a charge if that decoration isn't part of the charge by definition (e.g., a flowery bend), (3) any uncommon or unexpected object held or contained by an entity (e.g., a basket holding stars or a beaver eating a duck), (4) any pattern or motif superimposed on another: e.g., a checky lion.

- Any exceptional variant requiring a special mention in the database.

On the other hand, arms were retained when:

- They specified the motif's position on the shield: “per bend”, “per fess”, “in chief”, etc. (This did not apply to partitions or charges whose positions on the shield is fixed by definition).

- Their orientation was inverted.

- They specified an animal's posture: *rampant, courant, passant*, etc., even when that indication was not part of the motif's definition, as long as such modificatory were not issant or naissant (these terms indicate that only one half of an animal is shown). The adjectives *issant* and *naissant* were treated as introducing a different kind of motif, different from the whole animal, as Renesse usually treats such motifs.

When in doubt, we referred to Renesse's inventory of heraldic motifs, attempting to stay as close as possible to his classifications. All counts were made for each motif in English and in French to ensure robustness. In case of conflict, the French version was systematically preferred, since our reference classification (Renesse’s) is in French.

## **3.6. Frequency measures in the Renesse corpus**

In Renesse, the only arms to be counted exhaustively are the ones present in the “Armes complètes” section, meaning they are coats of arms bearing the relevant motif, and only this motif. Such arms were counted manually by a research assistant, systematically going through the volumes of Renesse’s inventory. Minor variations, signaled between parentheses in Renesse were counted in the focus motif’s occurrences, whereas more major variations, signaled by being mentioned in different paragraphs (or sub-paragraphs).

# **4. Results of analyses on the datasets without applying the exclusion criterion**

| Test | With exclusions (reported in the main text) | Without exclusions |
| --- | --- | --- |
| Correlation between perimetric and descriptive complexity | r_τ_ = .69, p < .001  95%CI = [0.657, 0.725] | r_τ_ = .70, p < .001  95%CI = [0.665, 0.729] |
| Difference in perimetric complexity between iconic and non iconic motifs | U = 32160, p < .01 | U = 34052, p < .01 |
| Difference in descriptive complexity between iconic and non iconic motifs | U = 36110, p < .01 | U = 38110, p < .01 |
| Overall correlation between perimetric complexity and frequency - Clemmensen corpus | r_τ_= -.02, p = .633  95%CI = [-0.103, 0.065] | r_τ_= -.03, p = .401  95%CI = [-0.116,0.049] |
| Overall correlation between descriptive complexity and frequency - Clemmensen corpus | r_τ_= -.09, p = .018  95%CI = [-0.177, -0.011] | r_τ_= -.11, p = .007  95%CI = [-0.189,-0.025] |
| Overall correlation between perimetric complexity and frequency - Renesse corpus | r_τ_ = .12, p < .001  95%CI = [0.058, 0.186] | r_τ_= .12, < .001  95%CI = [0.062,0.186] |
| Overall correlation between descriptive complexity and frequency - Renesse corpus | r_τ_ = .08, p = .008  95%CI=[0.02, 0.149] | r_τ_= .09, p = .004  95%CI = [0.026,0.153] |
| Correlation between perimetric complexity and frequency for non-iconic motifs only - Clemmensen corpus | r_τ_= -.08, p = .233  95%CI = [-0.211, 0.058] | r_τ_= -.07, p = .273  95%CI = [-0.204,0.063] |
| Correlation between descriptive complexity and frequency for non-iconic motifs only - Clemmensen corpus | r_τ_= -.14, p = .035  95%CI = [-0.27, -0.001] | r_τ_= -.13, p = .045  95%CI = [-0.262,0.005] |
| Correlation between perimetric complexity and frequency for non-iconic motifs only - Renesse corpus | r_τ_ = -.03, p = .534,  95%CI = [-0.147, 0.078] | r_τ_= -.04, p = .504  95%CI = [-0.148,0.075] |
| Correlation between descriptive complexity and frequency for non-iconic motifs only - Renesse corpus | r_τ_ = -.10, p = .07  95%CI = [-0.211, 0.011] | r_τ_= -.10, p = .066  95%CI = [-0.212,0.009] |
| Correlation between perimetric complexity and frequency for iconic motifs only - Clemmensen corpus | r_τ_= .12, p < .05  95%CI = [0.021, 0.226] | r_τ_= .09, p = .067  95%CI = [-0.01, 0.194] |
| Correlation between descriptive complexity and frequency for iconic motifs only - Clemmensen corpus | (r_τ_= .08, p = .14  95%CI = [-0.028, 0.18] | r_τ_= .05, p = .359  95%CI = [-0.057,0.149] |
| Correlation between perimetric complexity and frequency for iconic motifs only - Renesse corpus | r_τ_ = .22, p < .001  95%CI = [0.143, 0.291] | r_τ_= .21, p < .001  95%CI = [0.14,0.281] |
| Correlation between descriptive complexity and frequency for iconic motifs only - Renesse corpus | r_τ_ = .18, p < .001  95%CI = [0.108, 0.253] | r_τ_= .18, p < .001  95%CI = [0.111,0.253] |
| Change in frequency for iconic versus non-iconic motifs | U = 16004, p < .001 | U = 16962, p < .001 |
| Correlation between change in frequency and perimetric complexity | r_τ_ = .16, p < .001  95%CI = [0.076, 0.242] | r_τ_= .16, p < .001  95%CI = [0.08,0.242] |
| Correlation between change in frequency and descriptive complexity | r_τ_ = .22, p < .001  95%CI = [0.144, 0.297] | r_τ_= .22, p < .001  95%CI = [0.149,0.298] |
| Correlation between change in frequency and perimetric complexity - Iconic motifs only | r_τ_ = .11, p = .034  95%CI = [0.005, 0.206] | r_τ_= .11, p = .027  95%CI = [0.012,0.205] |
| Correlation between change in frequency and descriptive complexity - Iconic motifs only | r_τ_ = .08, p = .108  95%CI = [-0.011, 0.172] | r_τ_= .09, p = .067  95%CI = [0,0.179] |
| Correlation between change in frequency and perimetric complexity - Non-iconic motifs | r_τ_ = .03, p = .590  95%CI = [-0.105, 0.173] | r_τ_= .02, p = .710  95%CI = [-0.115,0.162] |
| Correlation between change in frequency and descriptive complexity - Non-iconic motifs | r_τ_ = .09, p = .161  95%CI = [-0.049, 0.227] | r_τ_= .08, p = .226  95%CI = [-0.061,0.214] |

1. Timestamped version can be found here : <https://osf.io/ct28b> [↑](#footnote-ref-1)
2. Timestamped registration can be found here: <https://osf.io/jwrsv> [↑](#footnote-ref-2)
3. Timestamped registration can be found here: <https://osf.io/b3pdt> [↑](#footnote-ref-3)
